# Next Class

**Published:** 1857-05

**Authors:** 


☞ From reliable information, we are enabled to say, that the approaching Class in the Atlanta Medical College, will show a large increase upon the last; they have already commenced assembling, though it is about two weeks previous to the commencement of the session.

